# Evaluating the feasibility of the KDIGO CKD referral recommendations

**DOI:** 10.1186/s12882-017-0646-y

**Published:** 2017-07-07

**Authors:** Karandeep Singh, Sushrut S. Waikar, Lipika Samal

**Affiliations:** 1 Division of Learning and Knowledge Systems, Department of Learning Health Sciences, University of Michigan Medical School, 1161H NIB, 300 N. Ingalls St, Ann Arbor, MI 48109-5403 USA; 2 Division of Nephrology, Department of Internal Medicine, University of Michigan Medical School, 1161H NIB, 300 N. Ingalls St, Ann Arbor, MI 48109-5403 USA; 3 Division of Renal Medicine, Brigham and Women’s Hospital, 75 Francis St, MRB4, Boston, MA 02115 USA; 4 Division of General Medicine, Brigham and Women’s Hospital, 1620 Tremont St Suite 03-02V, Boston, MA 02120 USA; 5 Harvard Medical School, Boston, MA USA

## Abstract

**Background:**

In 2012, the international nephrology organization Kidney Disease Improving Global Outcomes (KDIGO) released recommendations for nephrology referral for chronic kidney disease (CKD) patients. The feasibility of adhering to these recommendations is unknown.

**Methods:**

We conducted a retrospective analysis of the primary care population at Brigham and Women’s Hospital (BWH). We translated referral recommendations based upon serum creatinine, estimated glomerular filtration rate (eGFR), and albuminuria into a set of computable criteria in order to project referral volume if the KDIGO referral recommendations were to be implemented. Using electronic health record data, we evaluated each patient using the computable criteria at the times that the patient made clinic visits in 2013. We then compared the projected referral volume with baseline nephrology clinic volume.

**Results:**

Out of 56,461 primary care patients at BWH, we identified 5593 (9.9%) who had CKD based on albuminuria or estimated GFR. Referring patients identified by the computable criteria would have resulted in 2240 additional referrals to nephrology. In 2013, this would represent a 38.0% (2240/5892) increase in total nephrology patient volume and 67.3% (2240/3326) increase in new referral volume.

**Conclusions:**

This is the first study to examine the projected impact of implementing the 2012 KDIGO referral recommendations. Given the large increase in the number of referrals, this study is suggestive that implementing the KDIGO referral guidelines may not be feasible under current practice models due to a supply-demand mismatch. We need to consider new strategies on how to deliver optimal care to CKD patients using the available workforce in the U.S. health care system.

## Background

Several studies have identified benefits of early nephrology referral among CKD patients, including reduced mortality at 3 months to 5 years of follow-up [1, 2], reduced costs [3], reduced length of stay among hospitalized patients [1, 4], increased quality of life [2], and earlier placement of preferred dialysis access [1]. Early referral to a nephrologist has been recognized as a national priority. One objective of the U.S. Office of Disease Prevention and Health Promotion’s Healthy People 2020 initiative is to increase the proportion of chronic kidney disease patients receiving care from a nephrologist at least 12 months before the start of renal replacement therapy [5]. Based on mounting evidence favoring early referral, the international nephrology organization Kidney Disease Improving Global Outcomes (KDIGO) defined indications for nephrology referral as part of a broader guideline for CKD management in 2012 [6].

KDIGO defined indications for referral of CKD patients who are most likely to benefit from diagnostic work-up, close monitoring, or planning for renal replacement therapy through consultation with a nephrologist [4, 7, 8]. Referral to a nephrologist for this group of patients was considered a level 1B recommendation, denoting that it is based on “moderate” quality evidence. The indications for referral include acute kidney injury, progressive or advanced CKD, albuminuria, urinary red cell casts, refractory hypertension, potassium abnormalities, recurrent nephrolithiasis, and hereditary kidney disease.

The prevalence of CKD is over 10% [9] and the nephrology workforce is limited. While there are an estimated 246,500 primary care physicians currently practicing in the United States [10], the estimated practicing nephrology workforce is just over 9000 [11]. Referring all patients identified by the KDIGO CKD guidelines may not be feasible due to a mismatch in nephrologist supply and demand.

We sought to estimate the number of referrals that would be generated by adhering to the KDIGO referral recommendations in a primary care population among individuals with pre-existing CKD, and then compare this estimate against the baseline referral volume to assess the feasibility of adhering to these recommendations.

## Methods

### Participants

We first identified all patients seen in the Brigham and Women’s Hospital (BWH) primary care network of 15 practices from January 1 to December 31 of 2013. We then selected patients who had at least one visit dating back to 2009 with a BWH primary care to eliminate patients seen at BWH solely on a referral basis. Since KDIGO’s referral recommendations only apply to individuals with CKD, we then selected patients with CKD, defined as two estimated glomerular filtration rates (eGFRs) < 60 ml/min/1.73m^2^ or urine microalbumin/creatinine ratios ≥30 μg/mg at least 3 months apart at any time between 2007 and 2012. eGFR was calculated using the CKD-EPI formula [9].

Brigham and Women’s Hospital is a 793-bed tertiary care hospital that belongs to the Partners Healthcare system, the largest healthcare provider in Massachusetts. The BWH primary care network includes hospital-based practices, community-based practices, and two federally qualified health centers.

### Data source

We used retrospective patient data from the Partners Research Patient Data Registry (RPDR), which is the centralized clinical data warehouse for research at Partners Healthcare. From this source, we obtained two datasets. The first consisted of all patients seen in one of the 15 BWH primary care practices in 2013. The second consisted of all patients seen by a BWH nephrologist between 2005 and 2015. The first dataset was used to determine which patients met criteria for nephrology referral. The second dataset was used to measure nephrology outpatient practice volume, including patients referred to BWH nephrologists from non-BWH providers.

### Study design

We conducted a retrospective analysis to determine how adherence to the KDIGO referral recommendations would impact the number of nephrology referrals. Using the referral recommendations as a guide, we constructed a series of 12 computable criteria for which KDIGO recommends referral, focused on the following general indications: acute kidney injury (AKI), albuminuria, early CKD progression, late stable CKD, late CKD progression, and high annual decline in GFR (Table 1).

**Table 1:** Computable criteria for indications for nephrology referral in the KDIGO 2012 CKD guidelines

Diagnosis	Scenario	Criteria used to identify patients
Acute Kidney Injury	1	Creatinine rises by 0.3 mg/dL on most recent lab as compared to prior lab checks within last 48 h
	2	Creatinine has risen by at least 1.5 times within past 7 days
	3	Estimated GFR is 50% lower as compared to the baseline eGFR, with baseline eGFR defined as the highest eGFR from the past 6 months (“abrupt sustained fall in GFR”)
Late Stable Chronic Kidney Disease	4	Recent eGFR and eGFR prior to 3 months ago are both <30 ml/min/1.73m^2^
	5	Recent eGFR and eGFR prior to 3 months ago are both <15 ml/min/1.73m^2^
Albuminuria	6	Urine microalbumin/creatinine >300 μg/mg on last two consecutive checks at least 24 h apart
Chronic Kidney Disease Progression	7	eGFR drop from ≥90 (CKD stage 1) to <89 ml/min/1.73m^2^ (CKD stage 2), accompanied by a 25% drop in eGFR on most recent lab as compared to baseline, with baseline eGFR defined as the highest eGFR from the past year
	8	eGFR drop from 60 to 89 (CKD stage 2) to <59 ml/min/1.73m^2^ (CKD stage ≥3a), accompanied by a 25% drop in eGFR on most recent lab as compared to baseline, with baseline eGFR defined as the highest eGFR from the past year
	9	eGFR drop from 45 to 59 (CKD stage 3a) to <45 ml/min/1.73m^2^ (CKD stage ≥3b), accompanied by a 25% drop in eGFR on most recent lab as compared to baseline, with baseline eGFR defined as the highest eGFR from the past year
	10	eGFR drop from 30 to 44 (CKD stage 3b) to <30 ml/min/1.73m^2^ (CKD stage ≥4), accompanied by a 25% drop in eGFR on most recent lab as compared to baseline, with baseline eGFR defined as the highest eGFR from the past year
	11	eGFR drop from 15 to 29 (CKD stage 4) to <15 ml/min/1.73m^2^ (CKD stage 5), accompanied by a 25% drop in eGFR on most recent lab as compared to baseline, with baseline eGFR defined as the highest eGFR from the past year
High Annual GFR Decline	12	Mean eGFR drops ≥5 ml/min/1.73m^2^ per year, where the current year’s eGFR is defined by the mean eGFR for the last 365 days and the prior year’s eGFR is defined by the mean eGFR between 366 and 730 days ago

We focused on recommendations based upon serum creatinine, eGFR, and albuminuria because chronic kidney disease and acute kidney injury have well-accepted definitions in the nephrology community [12, 13] and can be readily computed using electronic health record (EHR) data. We did not include diagnoses such as hereditary kidney disease in the construction of the referral scenarios because electronic problem list documentation is known to be sparse [14] and billing codes may be inaccurate [15].

We then evaluated whether these patients met one of the KDIGO indications for referral at any of their outpatient visits in 2013. We only used information that would have been available to clinical providers on the date that each visit occurred. The definition of baseline eGFR or urine microalbumin/creatinine ratio varies among the 12 scenarios (Table 1) because the timing of the baseline value may appropriately differ in the setting of progressive chronic kidney disease and acute injury. We updated the baseline values at each visit based the definitions within each scenario. Patients were considered candidates for referral if they met criteria for referral during any of their visits in 2013. For patients to be considered “projected new referrals,” they had to meet both of these criteria: 1) qualify for any of the computable indications for nephrology referral during any of their outpatient clinical visits in 2013; and 2) have no outpatient or inpatient visits with a nephrologist in 2013.

To measure the impact of referral on current practice, we first determined baseline nephrology clinic volume at BWH in 2013. We then compared the number of patients who met criteria for each of the scenarios to the number who had either an outpatient visit or inpatient referral with a nephrologist in 2013 to determine the number of “projected new referrals” under the assumption of perfect guideline adherence.

Approval for this study was granted by the institutional review board at Partners Healthcare, and the need for informed consent was waived.

## Results

Out of 56,461 BWH primary care patients, we identified 5593 (9.9%) with pre-existing CKD. As compared to all primary care patients, the patients with CKD were older, less likely to be female, with a lower baseline eGFR and greater degree of albuminuria (Table 2).

**Table 2:** Baseline characteristics of primary care population and CKD population

Characteristic	Primary care population	All patients with CKD	*P*-value
No. of patients	56,461	5593	–
Age in years, mean (SD)	54.7 (15.5)	70.7 (12.4)	<0.001*
Female, n (%)	37,577 (66.6%)	3468 (62.0%)	<0.001**
Race			
Caucasian Black Hispanic Asian Other	35,323 (62.6%) 8612 (15.3%) 6900 (12.2%) 1599 (2.8%) 4027 (7.1%)	3488 (62.4%) 870 (15.6%) 806 (14.4%) 115 (2.1%) 314 (5.6%)	< 0.001**
Baseline eGFR, ml/min/1.73m^2^			
≥ 90 60–89 30–59 15–29 < 15 Not available	7386 (13.1%) 9860 (17.5%) 2888 (5.1%) 260 (0.5%) 121 (0.2%) 35,946 (63.7%)	309 (5.5%) 1572 (28.1%) 2600 (46.5%) 253 (4.5%) 120 (2.1%) 739 (13.2%)	< 0.001***
Urine microalbumin to creatinine ratio, μg/mg, median (IQR)	0 (IQR [0,35])	32.9 (IQR [0,131.1])	< 0.001***

Baseline eGFR is calculated based on the first available creatinine result in 2013

*Welch’s two-sample T-test

**Χ^2^ test

***Wilcoxon rank sum test

**Fig. 1:**
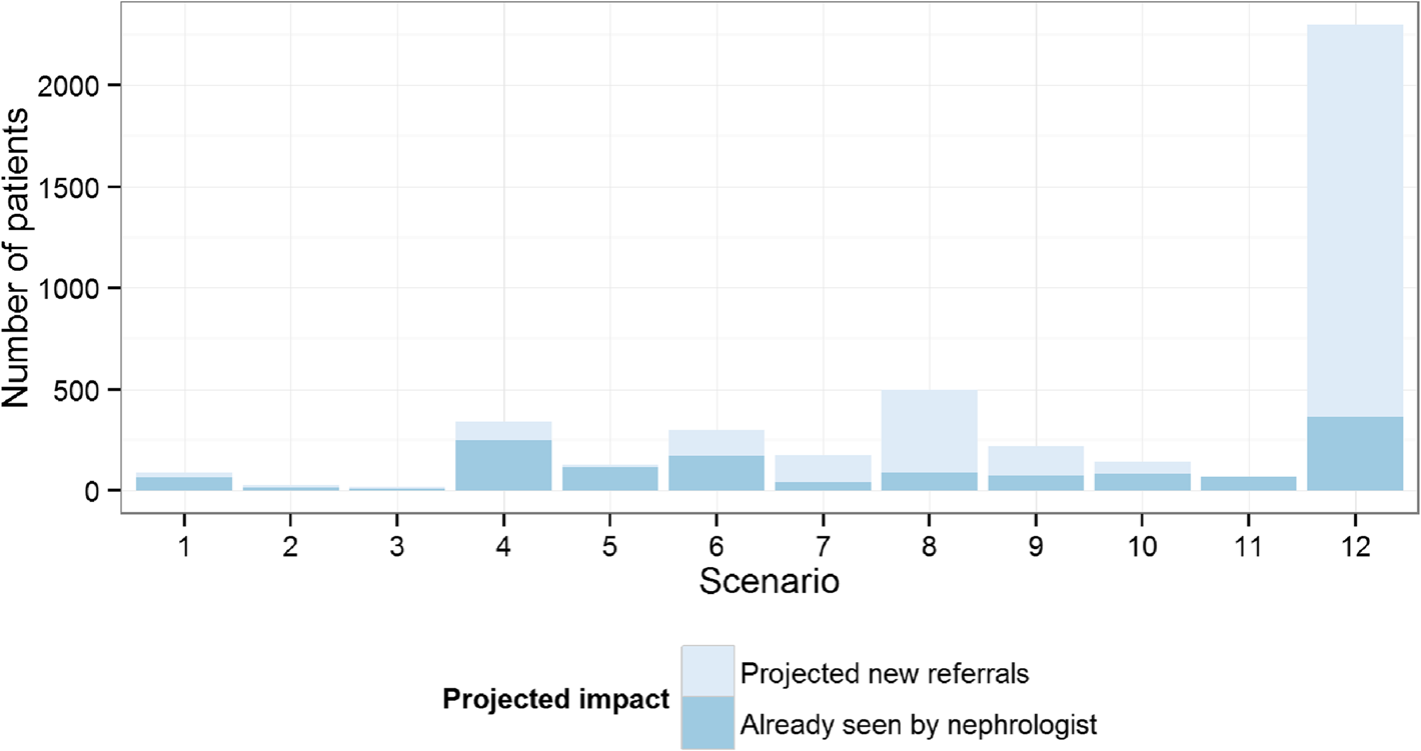
Comparison of projected referral volume with baseline referral volume in 2013 among patients with CKD meeting a referral indication

For the 5593 patients with pre-existing CKD, we evaluated 37,056 outpatient visits in 2013 and found that 2851 patients met criteria for at least one of the 12 computable scenarios for nephrology referral. Of these patients with CKD, only 21.4% (611/2851) were seen by a BWH nephrologist in 2013, while we classified the remaining 2240 as “projected new referrals” to nephrology (Fig. 1). In comparison to the CKD patients who saw a nephrologist in 2013, the projected new referrals were older, more likely to be female, more likely to be Caucasian and less likely to be black, and had a higher baseline eGFR and lesser degree of albuminuria (Table 3).

**Table 3:** Comparison of CKD patients meeting an indication for referral who were seen by a nephrologist in 2013 and projected new referrals

Characteristic	All patients with CKD meeting an indication for referral	Met indication and seen by nephrologist	Met indication and not seen by nephrologist (“projected new referral”)	*P*-value*
No. of patients	2851	611	2240	–
Age in years, mean (SD)	70.2 (12.7)	66.8 (13.8)	71.1 (12.2)	< 0.001***
Female, n (%)	1798 (63.1%)	349 (57.1%)	1449 (64.7%)	< 0.001****
Race				
Caucasian Black Hispanic Asian Other	1648 (57.8%) 539 (18.9%) 465 (16.3%) 63 (2.2%) 136 (4.8%)	291 (47.6%) 175 (28.6%) 105 (17.2%) 21 (3.4%) 19 (3.1%)	1357 (60.6%) 364 (16.3%) 360 (16.1%) 42 (1.9%) 117 (5.2%)	< 0.001****
Baseline eGFR, ml/min/1.73m^2^				
≥ 90 60–89 30–59 15–29 < 15 Not available **	147 (5.2%) 749 (26.3%) 1446 (50.7%) 233 (8.2%) 119 (4.2%) 157 (5.5%)	22 (3.6%) 72 (11.8%) 259 (42.4%) 130 (21.3%) 110 (18.0%) 18 (2.9%)	125 (5.6%) 677 (30.2%) 1187 (53.0%) 103 (5.6%) 9 (0.4%) 139 (6.2%)	< 0.001*****
Urine microalbumin to creatinine ratio, μg/mg, median (IQR)	41.5 (IQR [0,233.8])	133.5 (IQR [22.2,842.2])	29.1 (IQR [0,108.7])	< 0.001*****

Baseline eGFR is calculated based on the first available creatinine result in 2013

* *p*-value comparing characteristics for individuals seen and not seen by a nephrologist

** eGFR was not required to define CKD for individuals with persistent albuminuria

*** Welch’s two-sample T-test

**** Χ^2^ test

***** Wilcoxon rank sum test

To put this into perspective, the BWH nephrology clinic saw 5892 patients in 2013, of which 3326 were patients who were seen for the first time in 2013. If the 2240 additional patients meeting an indication for referral had been referred to a nephrologist in 2013, this would have resulted in a 38.0% (2240/5892) increase in total nephrology patient volume and 67.3% (2240/3326) increase in new referral volume.

Given the large number of projected new referrals attributable to a high annual decline in GFR (scenario 12), we carried out additional analyses to evaluate the extent to which this criterion was being met for early versus late CKD. Among the 1933 patients captured by this rule, 1274 (66%) had a mean eGFR <60 ml/min/1.73m^2^ in the prior year while the remaining 659 (34%) patients had a mean eGFR ≥60 ml/min/1.73m^2^ in the prior year.

## Discussion

This is the first study to examine the feasibility of adhering to the 2012 KDIGO referral recommendations. We found that 21.4% of all CKD patients meeting an indication for referral were actually referred to a nephrologist in 2013. Had the guidelines been followed, we found that 2240 additional patients, almost 4% of our primary care population, would have been referred to a nephrologist in 2013. Our finding that only a minority of individuals were referred upon meeting a referral indication is supported by data from the Healthy People 2020 initiative, which showed that only 33% of people were referred to a nephrologist at least 12 months before the start of renal replacement therapy in 2012 [5].

KDIGO recognizes that the guidelines may not be possible to implement in their stated form: “as a global guideline it is written for use in different health-care settings, but unavoidably its full implementation relies on health-care resources that are not universally available” [6]. However, if the guideline is not feasible to implement at a resource-rich tertiary care center, we question whether its implementation can be clearly fulfilled in any setting in the U.S. health care system. The density of nephrologists is likely to be higher in tertiary care centers than in community settings, so we would expect that it would be even less feasible to adhere to the KDIGO recommendations in community settings where nephrologists are in short supply.

In the face of limited nephrologist supply, several strategies may play a role in improving CKD care. Expanding the nephrology workforce may increase the capacity to provide specialty care for CKD patients but the workforce would need to grow by 38% to match the 38% growth in referrals, which is unlikely to be achievable. Another strategy is to optimize the management of CKD patients in primary care settings using health information technology. This may be achieved through computerized clinical decision support delivered at the point-of-care, delivery of appropriate interventions to a registry of patients using population health management software, and streamlining of co-management strategies between primary care physicians and nephrologist. Understanding how technology can be used to improve CKD care is a major focus area for the National Kidney Disease Education Program [16].

We also need to better understand why primary care physicians did not refer patients meeting one of the referral indications. It is possible that the guidelines have not been widely disseminated among primary care providers despite their being part of the target audience [6]. Prior surveys have shown that primary care providers and internal medicine trainees are unaware of both the presence and content of guidelines focused on CKD care [17–19]. It is also possible that primary care providers disagree with the guidelines for patients they decided not to refer. We found that projected new referrals were older than patients who were actually referred. Primary care physicians may have perceived this group to be inappropriate candidates for dialysis and thus opted not to refer them to a nephrologist.

One potential limitation is our translation of the guidelines to computable criteria and the exclusion of referral guidelines which are difficult to implement via computational techniques (e.g., hereditary kidney disease), which would mean that our estimate of new referrals is low. Our analysis is limited by its focus on a single tertiary care center’s affiliated primary care practices, which may be biased by local referral patterns. However, the BWH primary care population is diverse and drawn from a wide catchment area. We may have underestimated nephrology referrals due mischaracterization of provider specialty in the billing data or due to referrals by primary care providers to nephrologists outside our network; however, we randomly selected and reviewed 20 charts and did not find mischaracterization of provider specialty.

## Conclusions

The main implication of our findings is that adhering to the KDIGO referral recommendations may not be feasible. We need to consider new strategies on how to deliver optimal care to CKD patients using the available workforce in the U.S. health care system.

## Abbreviations

BWH: Brigham and Women’s Hospital
CKD: Chronic kidney disease
eGFR: Estimated glomerular filtration rate
EHR: Electronic health record
GFR: Glomerular filtration rate
KDIGO: Kidney Disease Improving Global Outcomes, an international nephrology organization
PCP: Primary care physician
